# Low-molecular-weight-heparin can benefit women with recurrent pregnancy loss and sole protein S deficiency: a historical control cohort study from Taiwan

**DOI:** 10.1186/s12959-016-0118-9

**Published:** 2016-10-28

**Authors:** Ming-Ching Shen, Wan-Ju Wu, Po-Jen Cheng, Gwo-Chin Ma, Wen-Chu Li, Jui-Der Liou, Cheng-Shyong Chang, Wen-Hsiang Lin, Ming Chen

**Affiliations:** 1 Department of Internal Medicine, Changhua Christian Hospital, Changhua, Taiwan; 2 Department of Obstetrics and Gynecology, Changhua Christian Hospital, Changhua, Taiwan; 3 Department of Genomic Medicine, Changhua Christian Hospital, 500 Changhua, Taiwan; 4 Department of Obstetrics and Gynecology, Chang-Gung Memorial Hospital Linkou Medical Center and Chang-Gung University, Taoyuan, Taiwan; 5 Department of Obstetrics and Gynecology, Puli Christian Hospital, Nantou, Taiwan; 6 Department of Obstetrics and Gynecology, Taipei Chang-Gung Memorial Hospital, Taipei, Taiwan; 7 Department of Obstetrics and Gynecology, and Department of Medical Genetics, College of Medicine, and Hospital, National Taiwan University, Taipei, Taiwan; 8 Department of Life Science, Tunghai University, Taichung, Taiwan

**Keywords:** Anticoagulation, Protein S deficiency, Thrombophilia, Recurrent miscarriages, Low-molecular-weight-heparin

## Abstract

**Background:**

Heritable thrombophilias are assumed important etiologies for recurrent pregnancy loss. Unlike in the Caucasian populations, protein S and protein C deficiencies, instead of Factor V Lieden and Prothrombin mutations, are relatively common in the Han Chinese population. In this study we aimed to investigate the therapeutic effect of low molecular weight heparin upon women with recurrent pregnancy loss and documented protein S deficiency.

**Methods:**

During 2011–2016, 68 women with recurrent pregnancy loss (RPL) and protein S deficiency (both the free antigen and function of protein S were reduced) were initially enrolled. All the women must have experienced at least three recurrent miscarriages. After excluding those carrying balanced translocation, medical condition such as diabetes mellitus, chronic hypertension, and autoimmune disorders (including systemic lupus erythematosus and anti-phospholipid syndrome), coexisting thrombophilias other than persistent protein S deficiency (including transient low protein S level, protein C deficiency, and antithrombin III), only 51 women with RPL and sole protein S deficiency were enrolled. Initially they were prescribed low dose Aspirin (ASA: 100 mg/day) and unfortunately there were still 39 women ended up again with early pregnancy loss (12 livebirths were achieved though). Low-molecular-weight-heparin (LMWH) was given for the 39 women in a dose of 1 mg/Kg every 12 h from the day when the next clinical pregnancy was confirmed to the timing at least 24 h before delivery. The perinatal outcomes were assessed.

**Results:**

Of 50 treatment subjects performed for the 39 women (i.e. 11 women enrolled twice for two pregnancies), 46 singletons and one twin achieved livebirths. The successful live-birth rate in the whole series was 94 % (47/50). Nineteen livebirths delivered vaginally whereas 28 delivered by cesarean section. The cesarean delivery rate is thus 59.57 %. Emergent deliveries occurred in 3 but no postpartum hemorrhage had been noted.

**Conclusions:**

Our pilot study in Taiwan, an East Asian population, indicated anti-coagulation therapy is of benefit to women with recurrent pregnancy loss who had documented sole protein S deficiency.

**Trial registration:**

ISRCTN64574169. Retrospectively registered 29 Jun 2016.

**Electronic supplementary material:**

The online version of this article (doi:10.1186/s12959-016-0118-9) contains supplementary material, which is available to authorized users.

## Background

Habitual abortion (defined as at least three recurrent miscarriages), namely recurrent pregnancy loss (RPL), is a condition caused by heterogeneous etiologies such as hormonal (luteal defect), chromosomal (carriers of balanced translocation), structural (Mullerian anomalies such as didelphys, bicornuate, or septate uterus), immunological (anti-phospholipid antibody or aberrations involving nature killer cells), and thrombophilia [1, 2]. Among them, heritable thrombophilias are treatable theoretically despite most published studies, including some but very limited well-conducted randomized trials, in the literature did not observe an apparent benefit by using anticoagulants to enhance the livebirth rate in women with RPL [3–9].

Heritable thrombophilias ever reported with clinical significance include protein S deficiency, protein C deficiency, anti-thrombin III deficiency, Factor V Leiden mutation, and prothrombin mutation, however, there are ethnic differences: The most common heritable thrombophilias in the Caucasian populations are Factor V Leiden mutation and prothrombin mutation whereas in Taiwan, protein S, protein C, and antithrombin III deficiencies are the most common [10–14]. Despite most published reports in the literature failed to observe a beneficial effect of anti-coagulants to enhance the livebirth rates in women with RPL, it may be inappropriate to extrapolate those results, mainly based on other ethnic groups, into an East Asian population (Taiwan is a multi-ethnic group country with a predominance of Han Chinese). According to our previous studies, the most common heritable thrombophilias in the thromboembolic patients in Taiwan are protein S and protein C deficiencies [13, 14]. Meanwhile, the selection criteria and treatment protocol varied across different studies and thus we should be cautious when reading reviews based upon meta-analysis [3–5, 15].

We are keen to explore if there is any role of using anticoagulants in this group of patients in Taiwan. In order to better define the enrollment criteria, those with other confounding factors such as anti-phospholipid antibody syndrome, underlying medical conditions including autoimmune diseases, diabetes mellitus, chronic hypertension, and previous history of thromboembolism are excluded. Only nulliparous women with protein S deficiency and suffered from RPL are enrolled in this pilot study. We intended to include women with in whom protein S deficiency is the only attributable etiology, and from another point of view, to include women with protein S deficiency who were otherwise healthy (that is, without previous thromboembolic events) until adult life except suffering from RPL. In addition, in order to simplify and better understand the actual effect of anti-coagulants in the enrolled cases, we only included those receiving low-molecular-weight-heparin as the sole therapy. The livebirth rate is the primary outcome we aimed to observe in this historical cohort. Attributable causes of the failed cases, if any, will be assessed by examinations including fetal/placental pathology, immunological and genetic investigations.

## Methods

### Patient enrollment

During 2011–2016, women suffered from at least three recurrent miscarriages who came to our clinic received a series of investigation including karyotyping, thrombophilia profile (antithrombin III, protein C, Protein S levels), and immune profile (lupus anti-coagulant to explore if she has anti-phospholipid antibody syndrome). Only those women with sole protein S deficiency, and naturally conceived, were enrolled. They were prescribed low dose Aspirin (ASA, 100 mg per day) when another new pregnancy was achieved and in those ended up again with early pregnancy loss (less than 12 complete weeks of gestation), these patients were enrolled in this cohort and would be given daily anti-coagulant treatment in the injection starting from when next pregnancy is established. It is noteworthy that since the level of protein S may decrease physiologically during pregnancy due to the estrogen effect, only those with persistent low protein S level in the non-gestational period (we enrolled only those with low levels in both protein S function and free protein S antigen (Ag)), were included. Assays adopted for protein S measurement used the functional assay by clotting based kits (the reference ranges: protein S function 63.5~149 %; free protein S antigen: 54.7~123.7 %) according to the method reported by Moraes and colleagues in 2000 [16]. Women with other heritable thromobophilias (Protein C and antithrombin III deficiency), immune problems (such as systemic lupus erythematosus, anti-phospholipid antibody syndrome), other pre-conceptional underlying medical conditions (such as diabetes mellitus and chronic hypertension), or carrying balanced translocation, were excluded. The institutional review board (IRB) of Changhua Christian Hospital had approved the study (CCH-IRB-151209).

### Clinical management protocol

Low-molecular-weight-heparin (enoxaparin) was given in a dose of 1 mg/Kg every 12 h from the day being enrolled to the timing a few days (at least 24 h) before delivery. Standard antenatal care was unaffected otherwise and the perinatal outcome was assessed. The patients received both the antenatal care from two of the coauthors (M Chen, an obstetrician who specializes in high-risk pregnancy, and MC Shen, a hematologist who specializes in thrombosis and hemostasis). In order to avoid the risk of postpartum hemorrhage due to emergent deliveries, which often occurs unexpectedly, the patients were either arranged for induction of labor if she chose to deliver vaginally, or scheduled cesarean section if there are obstetric indications or by their autonomous choice to choose elective cesareans. The anticoagulants were ought to be stopped at least 24 h before delivery in the ideal situation. The risk of postpartum hemorrhage as well as other side effects that may occur because of this treatment protocol were discussed and explained in great details to these patients and the informed consents were obtained. The blood coagulation profiles such as prothrombin time (PT), activated partial thromboplastin time (aPTT), or other assays to assess the status of the molecules involving the coagulation cascade were not regularly monitored. The livebirth rate is the primary outcome indicator. Secondary outcome indicators are also recorded for the obstetric complications such as premature births, low birth weight, and pre-eclampsia. The whole investigation period reported in this historical control cohort study ended at 22, May, 2016.

## Results

**Fig. 1:**
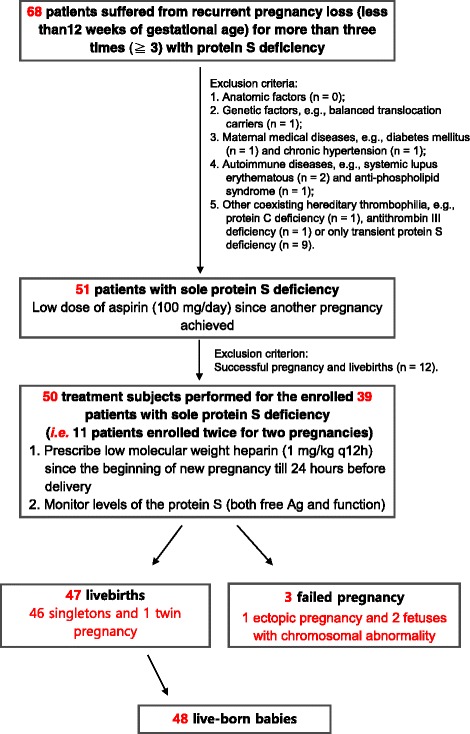
Patient summary and treatment flowchart

A total of 68 patients with at least three recurrent miscarriages and having protein S deficiency (both the free antigen and the function of protein S) went to our clinics. In these 68 patients, 17 of them were further filtered out because these patients were found with heritable thromobophilias other than protein S deficiency (protein C deficiency (*n* = 1), antithrombin III (*n* = 1), immune problems (e.g., systemic lupus erythematosus (*n* = 2), anti-phospholipid antibody syndrome (*n* = 1)), or with other underlying medical conditions (e.g., diabetes mellitus (*n* = 1) and chronic hypertension (*n* = 1)), or carrying balanced translocation (*n* = 1). Another 9 women who only had transient low level of protein S during pregnancy were also excluded. Therefore, 51 women entered the cohort initially (12 of them enjoyed livebirths simply by being given low dose Aspirin in a dose with 100 mg per day). Of the remaining 39 women, 11 had two pregnancies being enrolled. Consequently, 50 treatment subjects were performed for the 39 women and 47 livebirths were achieved (Fig. 1). Only 3 pregnancies failed, two of which were later proved to be an aneuploidy pregnancy (trisomy 22 and monosomy X respectively) and one was ectopic pregnancy ended with tubal abortion. Of the 11 women with two pregnancies enrolled, 10 had successful two livebirths and one had one livebirth with one abortion due to trisomy 22. In 47 successful livebirths, 48 live babies were born (because there were one twin pregnancy), 19 of them delivered vaginally (40.43 %), and 28 of them delivered by cesarean section (59.57 %). Notably only 4 of the cesarean group demanded elective cesarean section due to the concern about the risk of natural births, and all the remaining 24 out of the 28 women in the cesarean group had obstetric indications. The indications include previous uterine surgery (*n* = 5, two are both repeated sections and entered the group twice and the first cesareans were elective), placenta previa (*n* = 2), prolonged labor (*n* = 2), fetal malpresentation (*n* = 6, one case is twin pregnancy with one fetal malpresentation), non-reassuring fetal heart rate tracings (*n* = 8), and severe pre-eclampsia (*n* = 1). All the successful live babies were born after gestational age (GA) 28 weeks (Table 1), with the patient numbers (percentage) delivered at GA 36 weeks or greater, between GA 32 and 35^+6^ weeks, between GA 28 and 31^+6^ weeks and less than 28 weeks were 33 (70.21 %), 12 (25.23 %), 2 (4.26 %), and 0 (0 %). The distribution of the percentiles (calculated by Hadlock chart) regarding birth body weights of these babies was 27.08 % (*n* = 13, < 10th percentile, “small for gestational age (SGA)” by definition), 41.67 % (*n* = 20, 10th–25th percentile), 22.92 % (*n* = 11, 25th–50th percentile), and 8.33 % (*n* = 4, > 50th percentile) (Table 2). Nine of the babies born with SGA were in the cesarean section group (exactly those whose indications for cesareans were non-reassuring fetal heart rate tracings (*n* = 8) and severe-preeclampsia (*n* = 1)). Only 3 of the entire cohort delivered emergently (two vaginal births and one cesarean birth) and no postpartum hemorrhage occurred. Pathological examination of the placentae belonging to the SGA babies all showed gross hypoplasia and histological vascular lesions.

**Table 1:** Summary of the gestational age (GA) at delivery and mode of delivery of the 47 livebirths

	% (= *n*/47)
GA at delivery	
≥ 36 weeks	70.2 (33/47)
32–35^+6^ weeks	25.23 (12/47)
28–31^+6^ weeks	4.26 (2/47)
< 28 weeks	0 (0/47)
Mode of delivery	
Normal spontaneous delivery	40.43 (19/47)
Cesarean section	59.57 (28/47)

**Table 2:** Percentile distributions of the birth body weight of the 48 live-birth babies

Percentile of birth body weight (Hadlock)	% (= *n*/48)
SGA (<10th percentile)	27.08 (13/48)
10th–25th percentile	41.67 (20/48)
25th–50th percentile	22.92 (11/48)
>50th percentile	8.33 (4/48)

*SGA* small for gestational age

In summary, the rate of successful livebirth was thus 94 % (47/50). If by comparing to their historical cohort (the livebirth rate before being enrolled into the treatment group is theoretically zero) themselves, the benefit to this group of patients is clear (*p* = 0 by Fisher’s exact test).

## Discussion

Recurrent pregnancy loss (RPL), or recurrent miscarriages, is a serious problem in women’s health. It affects 1–2 % of women of reproductive age if 3 or more first trimester pregnancy losses (less than 12 complete gestational weeks) and 5 % of women of reproductive age if 2 or more first trimester pregnancy losses (less than 12 complete gestational weeks) [7]. The causes of RPL are varying and complicated. Etiologies that had been reported included structural (e.g., mullerian anomalies), hormonal (e.g., luteal defect), chromosomal (e.g., balanced translocation carriers), immune-related (e.g., anti-phospholipid antibody syndrome), thrombophilias (e.g., factor V Lieden mutations and prothrombin mutations in the caucasian populations), and others. Strategies to combat these putative causative factors were therefore developed, with varying efficacies [17, 18]. For example, some advocated anti-coagulation therapy (such as aspirin or low-molecular-weight-heparin) is effective in improving the pregnancy outcome in women suffered from anti-phospholipid antibody syndrome and RPL but the results were inconsistent among different trials [6, 19–21]. Most of the recently conducted randomized trials failed to show significant benefit of anti-coagulation therapy (either aspirin or low molecular weight heparin alone or if both were combined) to improve the livebirth rates in women with RPL [6, 7, 15]. However, these well-conducted trials actually did not well characterize the underlying possible causative factors in their study subjects, the subgroups due to different etiologies were pooled and no specific analyses were conducted separately on each subgroup.

There are at least three randomized trials conducted in earlier times dealing with women with RPL and a concomitant heritable thrombophilia and the results were varying. In 2004, the French group published their result of the randomized trial to compare low-molecular-weight heparin (LMWH) versus aspirin (ASA) and reported that LMWH was superior to ASA regarding the livebirth rates. In 2008, the Jordan group reported a similar beneficial effect of anticoagulant therapy (LMWH versus placebo, please refer to Qubian et al., 2008 [22]). However, the Canadian group reported there seemed no benefit to the livebirth rates when comparing LMWH plus ASA and ASA alone in 2009 (the HepASA Trial, please refer to Laskin et al., 2009 [23]). Tan and colleagues therefore conducted a meta-analysis and concluded that no obvious benefit can be attributed to the anti-coagulation therapy regarding the improvement of livebirth rates in women with RPL and a heritable thrombophilia [5]. However, the mixture of those three randomized trials is too arbitrary to exclude the possibility of real benefit in this group of women since the three randomized trails had different inclusion criteria and even different therapeutic regimens.

It is noteworthy that there are some recently well-conducted randomized trials being published and all of these trials failed to demonstrate the benefit of anticoagulant therapy in women with RPL (but not aiming solely at women with heritable thrombophilias). The Netherland study published in NEJM 2010 did included some women with heritable thrombophilas (even included protein S deficiency) but the authors also admitted the study lacked the power to study the effect in the subgroups of the study population [6]. The Scottish Pregnancy Intervention study (SPIN) also included some women with heritable thrombophilia but admitted their result did not exclude the possible benefit in women with a particular thrombophilia disorder as well [7]. A recent multi-center with minimized randomization scheme conduced in Germany and Austria actually excluded the women with constitutional thrombophilia disorders and therefore the trial result is of no reference value to our study [15].

In our study, it is obvious LMWH is beneficial to the livebirth rate in women suffered from RPL with documented sole protein S deficiency (94 versus 0 % by historical control). Particularly, 2 of the 11 women treated twice had initially decided not being enrolled into the treatment group receiving anticoagulants after one successful birth and got pregnant again and both of them suffered from spontaneous abortion (the karyotyping results of the abortion were both normal), and thereby they entered the study for the second time when again getting pregnant and successful livebirths ensued. Such experience strengthened the justification of this study. However, it seems LMWH was unable to prevent other obstetric complications such as placental insufficiency and therefore intrauterine growth restriction (IUGR) are common in our cohort. Among 48 live born babies, Nearly 70 % of them were born with birth weight less than 25th percentile (68.75 %; *n* = 33), and the percentage of small for gestational age (SGA) was 27.08 % (*n* = 13). Among the babies born with SGA, five infants were born before 36 weeks of GA, including one case of severe pre-eclampsia, one case of oligohydroamnios, and all of them demonstrated non-reassuring fetal heart rate tracings during intrapartum. The result was compatible with the prior reports [24], showed a link between thrombophilias and fetal growth restriction (odds ratio (OR) 10.2; 95 % confidence interval (CI) 1.1–91.0) by meta-analysis. However, available evidences in the literature do not support the prophylactic use of anticoagulants can prevent obstetric complications, including preeclampsia, fetal growth restriction, or abruption in women with any form of inherited thrombophilias [25], despite it may of be benefit in women with recurrent implantation failures when receiving in vitro fertilization [26]. It is still under debate if thrombophilias truly associated, or simply it is only by chance a coincidence, with the placental insufficiency [27]. Further investigations and surveys were required to establish causality. Hence, in addition to anti-coagulation therapy, frequent fetal surveillance is necessary for pregnant women with thrombophilia to prevent adverse neonatal outcomes. The American Congress of Obstetricians and Gynecologists (ACOG) did not suggest special management in thrombophilia patients in the absence of obstetric complications such as preeclampsia, abruption or IUGR. Weekly fetal assessment with non-stress test beginning at ≥36 weeks of gestation and delivery at 39 weeks of gestation is still the recommended standard of care when managing these patients [28].

We admit the evidence level of historical control is much lower than prospective randomized control trials. Many inherent defects underlying the historical control study hampered its use. The most frequently cited defects of this historical control conducted by patients as their own controls included different diagnostic criteria, differences in the concomitant standard of care, and missing records across different times along the time period (especially if the time spans very long such as 10 or 20 years). However, the credibility of historical control study may be better if there is a large treatment effect, or it is very difficult to bias outcome assessment, or it follows the pair availability design (proposed by Baker and Lindeman in 1994) [29–32].

## Conclusion

In this study, a strikingly high 94 % successful livebirth rate, straight-forward outcome indicators (livebirth rate and pregnancy complications), and the short time period (within 5 years), all indicated that our result is of better power, despite the limitations of historical control discussed above, to convince us that such intervention did benefit this group of patients in Taiwan. Our study clearly demonstrated a potential benefit of daily anti-coagulation therapy in women suffered from RPL and had documented sole protein S deficiency. Future prospective randomized studies are needed to further prove its efficacy.

## Additional file

Additional file 1: Table S1. Details of the 47 livebirths (resulting in 48 live-birth babies from 46 singletons and 1 twin pregnancy) from 37 patients prescribed low molecular weight heparin low molecular weight heparin. (XLSX 15 kb)

## Abbreviations

ACOG: The American Congress of Obstetricians and Gynecologists
ASA: Aspirin
CI: Confidence interval
GA: Gestational age
IUGR: Intrauterine growth restriction
LMWH: Low-molecular-weight heparin
OR: Odds ratio
RPL: Recurrent pregnancy loss
SGA: Small for gestational age
